# Paper Withdrawn Before the Issue Release

**DOI:** 10.3390/s120100878

**Published:** 2012-01-31

**Authors:** Shu-Kun Lin

**Affiliations:** MDPI AG, Postfach, CH-4005 Basel, Switzerland; Office: Kandererstrasse 25, 4057 Basel, Switzerland

The following paper “Juan V. Capella, Alberto Bonastre, Miguel Peris and Rafael Ors. Distributed In-Line Analysis of Water Pollution in a Spanish Lake.*Sensors***2012**, *12*, 878–894” has been withdrawn at the request of the authors before the issue release of *Sensors* Volume 12, Issue 1. We apologize for any inconvenience this may cause.

